# Unveiling the Role of *GhP5CS1* in Cotton Salt Stress Tolerance: A Comprehensive Genomic and Functional Analysis of *P5CS* Genes

**DOI:** 10.3390/plants14020231

**Published:** 2025-01-15

**Authors:** Hui Fang, Xin Gao, Yunhao Wu, Ke Zhang, Ying Wu, Junyi Li, Dongmei Qian, Ruochen Li, Haijing Gu, Teame Gereziher Mehari, Xinlian Shen, Baohua Wang

**Affiliations:** 1 Institute of Modern Agriculture, School of Life Sciences, Nantong University, Nantong 226019, China; fanghui8912@126.com (H.F.); 19963272263@163.com (X.G.); wuyunhao0323@126.com (Y.W.); abc3315481873@163.com (K.Z.); 15312928136@163.com (Y.W.); 15358302605@163.com (J.L.); 15951301606@163.com (D.Q.); liruochen2663@163.com (R.L.); 15936135237@163.com (H.G.); fiamieta21@gmail.com (T.G.M.); 2 State Key Laboratory of Cotton Bio-Breeding and Integrated Utilization, Institute of Cotton Research of Chinese Academy of Agricultural Sciences, Anyang 455000, China; 3 Key Laboratory of Cotton and Rapeseed (Nanjing) of the Ministry of Agriculture, The Institute of Industrial Crops, Jiangsu Academy of Agricultural Sciences, Nanjing 210014, China

**Keywords:** cotton genomics, *P5CS* gene family, *GhP5CS1* function, salt stress, proline, virus-induced gene silencing (VIGS)

## Abstract

Proline, a critical osmoregulatory compound, is integral to various plant stress responses. The *P5CS* gene, which encodes the rate-limiting enzyme in proline biosynthesis, known as ∆1-pyrroline-5-carboxylate synthetase, is fundamental to these stress response pathways. While the functions of *P5CS* genes in plants have been extensively documented, their specific roles in cotton remain inadequately characterized. In this study, we identified 40 *P5CS* genes across four cotton species with diverse sequence lengths and molecular weights. Phylogenetic analysis of 100 *P5CS* genes from nine species revealed three subgroups, with *Gossypium hirsutum* closely related to *Gossypium barbadense*. Collinearity analysis highlighted significant differences in collinear gene pairs, indicating evolutionary divergence among *P5CS* genes in tetraploid and diploid cotton. Exon–intron structures and conserved motifs correlated with phylogenetic relationships, suggesting functional differentiation. Stress-responsive elements in *P5CS* promoters suggest involvement in abiotic stress. Expression analysis under salt stress revealed differential expressions of *GhP5CS* genes, with *GhP5CS1* emerging as a potential key regulator. Virus-induced gene silencing confirmed the pivotal role of *GhP5CS1* in cotton’s salt stress response, as evidenced by increased salt sensitivity in the silenced plants. This study enhances our understanding of the functional diversity and roles of *P5CS* genes in cotton under stress conditions.

## 1. Introduction

Proline, characterized by its unique and multifunctional properties, plays a crucial role in various biological processes. Its distinctive side chain structure contributes to the structural stability and functionality of proteins, rendering it an indispensable element in protein structure and activity [1,2]. Additionally, proline functions as a compatible solute and a reactive oxygen scavenger, thereby shielding plants from oxidative stress. Consequently, proline is integral to plant growth and development, particularly in response to environmental stresses. The accumulation of proline in plants can be triggered by a range of abiotic and biotic stresses, including salinity [3,4,5], drought [6,7], ultraviolet (UV) radiation [8,9], heavy metal exposure [10], and various biotic challenges [11,12], among others.

Proline biosynthesis occurs through two distinct pathways: one that derives from glutamic acid and the other from ornithine. In the glutamate pathway, the initial two enzymatic reactions are catalyzed by the bifunctional enzyme P5CS, which exhibits both γ-glutamyl kinase and glutamic-γ-semialdehyde dehydrogenase activities [13]. P5CS represents the rate-limiting step in proline biosynthesis and is pivotal in regulating this metabolic process [13,14]. An upregulation of *P5CS* gene expression can effectively enhance proline accumulation, thereby augmenting the capacity of plants to withstand stresses [15,16,17]. The glutamic acid pathway predominantly facilitates proline accumulation under stress conditions, whereas the ornithine pathway serves as an alternative route that primarily operates under conditions of adequate nitrogen availability and does not participate in proline accumulation during stress.

Given the critical biological functions of proline, there has been a increasing amount of research focused on the role of *P5CS* genes, particularly in the context of abiotic stress regulation across various plant species. Numerous strategies have been employed to enhance plant stress resistance through the manipulation of the *P5CS* gene to promote proline accumulation. For instance, the overexpression of *NtP5CS*, a *P5CS* gene derived from the halophyte *Nitraria tangutorum*, has been shown to improve growth in *Escherichia coli* under diverse conditions [18]. Furthermore, the microRNA *ath*-*miR164c* has been identified to be a regulator of pathogen resistance and drought tolerance in *Arabidopsis thaliana* by modulating *AtP5CS1* expression and proline levels; in a loss-of-function mutant of *ath*-*miR164c*, both *AtP5CS1* expression and proline content were elevated compared to the control under combined stress conditions [11]. In wheat, lines overexpressing *TaP5CS1* exhibit increased proline levels and enhanced drought tolerance relative to wild-type plants [19]. Similar findings have been reported in various other species, including the loss-of-function mutant of *Mtp5cs3* in *Medicago truncatula* under saline conditions [20], the ectopic overexpression of the *LpP5CS* gene (*Lolium perenne* L.) in switchgrass (*Panicum virgatum* L.) under salt stress [21], the ectopic overexpression of *SpP5CS* (*Stipa purpurea*) in *A. thaliana* under drought conditions [22], and the ectopic overexpression of *PgP5CS* (*Pennisetum glaucum*) in tobacco under heat stress [23]. Collectively, these studies underscore the essential role of *P5CS* genes in mediating plant responses to environmental stresses.

Gene family analysis is essential for deciphering the functional roles and regulatory mechanisms associated with specific genes. To date, members of the *P5CS* gene family have been characterized in various species [15,24,25,26]; however, there is limited information regarding this gene in cotton. Cotton is a crop of significant economic importance globally, primarily due to its soft fibers, which are vital to the textile industry. The phenomenon of soil salinization presents a considerable challenge to cotton cultivation, negatively impacting growth and productivity by inducing osmotic and ionic imbalances within the plants [27,28]. Consequently, this study undertook a comprehensive analysis of the *P5CS* gene family across four cotton species: *Gossypium hirsutum* (hereafter *G. hirsutum*), *Gossypium arboretum* (*G. arboretum*), *Gossypium raimondii* (*G. raimondii*), and *Gossypium barbadense* (*G. barbadense*). The analysis encompassed phylogenetic evolution, gene structure, cis-acting elements, expression profiles in response to salt stress treatment, and functional validation through gene silencing mediated by VIGS. The results of this study will contribute to a deeper understanding of the regulatory roles of *P5CS* genes in response to stress in cotton.

## 2. Results

### 2.1. Identification and Sequence Analysis of the P5CS Gene Family in Cotton

Following a rigorous screening process using SMART, NCBI-CDD, and Pfam databases, a total of 40 *P5CS* genes were identified across four cotton species in the cotton Phytozome database. Specifically, *G. hirsutum* harbored 8 genes, *G. barbadense* contained 18 genes, *G. raimondii* contained 9 genes, and *G. arboreum* included 5 genes. These genes were designated as *GhP5CS1*–*GhP5CS8*, *GbP5CS1*–*GbP5CS18*, *GrP5CS1*–*GrP5CS9*, and *GaP5CS1*–*GaP5CS5*, respectively, based on their physical positions on the chromosomes (Supplementary Figure S1). The distribution of these genes was uneven, with a majority localized to both ends of each chromosome, and each chromosome typically hosts only one or two genes. Among the eight *GhP5CS* genes, four genes were mapped to four chromosomes of the A subgenome, namely A01, A07, A08, and A09, whereas the remaining four genes were found on four chromosomes of the D subgenome, namely D01, D04, D07, and D10. We subsequently analyzed the physical and chemical properties, as well as the sequences, of all identified *P5CS* genes (Supplementary Table S1). The 40 P5CS proteins exhibited varying sequence lengths, from 313 amino acids in GbP5CS17 to 916 amino acids in GrP5CS4, with corresponding molecular weights ranging from 33,494 Da (GbP5CS17) to 99,854.2 Da (GrP5CS4), which suggested a positive correlation between protein length and molecular weight. These findings indicate potential variations in protein sequences, domains, and expression patterns, which may underlie the functional diversity. Furthermore, the theoretical isoelectric points (PIs) of the identified P5CS proteins spanned from 5.87 to 9.61, with the majority being weakly acidic and a few being weakly alkaline (Supplementary Table S1). This further indicates functional differentiation among the P5CS proteins.

### 2.2. Evolutionary and Selection Analysis of P5CS Genes in Cotton

**Figure 1:**
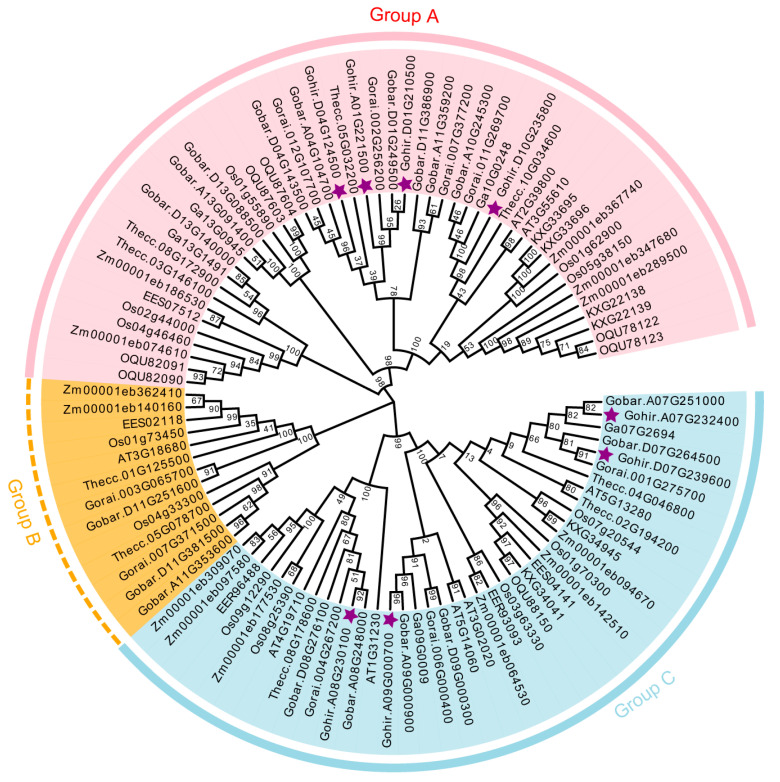
Phylogenetic analysis of *P5CS* genes across nine species. The depicted phylogenetic tree includes representatives from six dicotyledonous species (*G. hirsutum*, *G. barbadense*, *G. raimondii*, *G. arboretum*, *A. thaliana*, and *T. cacao*) and three monocots (*O. sativa*, *Z. mays*, and *S. bicolor*). The tree was constructed using MEGA 7.0 software with the maximum likelihood (ML) method. It is clustered into three distinct subgroups, each highlighted by a unique background color. *GhP5CS* genes are indicated with purple asterisks.

To elucidate the evolutionary relationships among *P5CS* genes in both monocotyledonous and dicotyledonous plants, we constructed a phylogenetic tree using protein sequences from nine species. It contains six dicot species, such as *G. hirsutum* (8 members), *G. barbadense* (18 members), *G. raimondii* (9 members), *G. arboretum* (5 members), *A. thaliana* (8 members), and *Theobroma cacao* (9 members), as well as three monocot species, namely *Oryza sativa* (12 members), *Zea mays* (13 members), and *Sorghum bicolor* (18 members) (Figure 1). A total of 100 P5CS proteins were categorized into three subgroups, designed as Groups A to C. Group A comprised 47 members, Group B had 13 members, and Group C included 40 members. The P5CS proteins in *G. hirsutum* were evenly distributed in Groups A and C, with four members in each. Phylogenetic analysis revealed that *P5CS* genes in cotton are closely related to the evolutionary branches of monocot plants, suggesting potential functional divergency between monocots and dicots. Furthermore, the *P5CS* genes in *G. hirsutum* tended to cluster with those in the tetraploid *G. barbadense* on the phylogenetic tree, indicating a closer evolutionary relationship.

Collinearity analysis effectively reflects gene homology, and collinear homologous genes are likely to share similar functions. We analyzed the interchromosomal collinearity of *P5CS* genes across the genomes of four cotton species. In *G. hirsutum*, we identified five collinear gene pairs involving six *GhP5CS* genes. In *G. barbadense*, 15 collinear gene pairs related to 14 *GbP5CS* genes were detected, primarily between homologous chromosomes. Additionally, five and one collinear gene pairs were identified in the genomes of *G. raimondii* and *G. arboretum*, respectively (Supplementary Figure S2). The presence of collinear gene pairs within a species suggests evolutionary events such as gene duplication and chromosomal rearrangements, potentially leading to functional divergence among gene family members.

**Figure 2:**
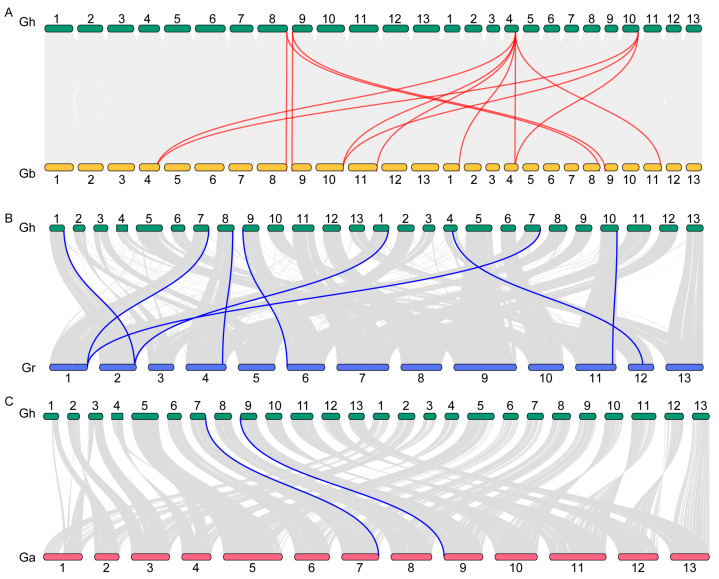
Collinearity of *P5CS* genes between *G. hirsutum* and three other cotton species: *G. barbadense*, *G. raimondii*, and *G. arboretum*. (**A**) Collinearity analysis of *P5CS* genes between *G. hirsutum* and *G. barbadense*. (**B**) Collinearity analysis of *P5CS* genes between *G. hirsutum* and *G. raimondii*. (**C**) Collinearity analysis of *P5CS* genes between *G. hirsutum* and *G. arboretum*. The gray lines in the background delineate genome-wide collinear blocks, while the red and blue lines specifically denote the collinearity of *P5CS* genes, revealing evolutionary relationships and genomic conservation among these species.

To further investigate the evolutionary relationships of *P5CS* genes, we performed collinearity analysis of *P5CS* genes between *G. hirsutum* and three other cotton species, namely *G. barbadense*, *G. raimondii*, and *G. arboretum*. We found significant differences in collinear gene pairs between tetraploid *G. hirsutum* and *G. barbadense*, and between tetraploid *G. hirsutum* and the two diploid cotton species (Figure 2). Specifically, thirteen collinear gene pairs were found between *G. hirsutum* and *G. barbadense*, while eight and two collinear gene pairs were found between *G. hirsutum* and *G. raimondii*, and for *G. arboretum*, respectively (Supplementary Table S3). These results suggest substantial evolutionary divergence of *P5CS* genes between tetraploid and diploid cotton species. The high number of collinear gene pairs between *G. hirsutum* and *G. barbadense* indicates a closer genetic relationship, suggesting that they have undergone less genetic divergence since their divergence from a common ancestor, maintaining a greater degree of synteny. In contrast, a low number of collinear gene pairs (only three) were found between two diploid species, *G. raimondii* and *G. arboretum*, indicating a more distant evolutionary relationship (Supplementary Figure S3 and Table S2).

To explore the selection events that the *P5CS* genes have undergone during evolution in cotton, we analyzed the selection pressure by calculating the KA, KS, and their ratio of *P5CS* gene pairs. Across the four cotton species, we verified one to fifteen homologous pairwise genes, and their KA/KS ratios ranged from 0.046 to 0.221 (Supplementary Table S3). These findings indicate that these *P5CS* genes were under purifying selection during evolution, with natural selection maintaining protein integrity by eliminating harmful mutations.

### 2.3. Gene Structure and Conserved Motif Analysis of P5CS Genes in Cotton

**Figure 3:**
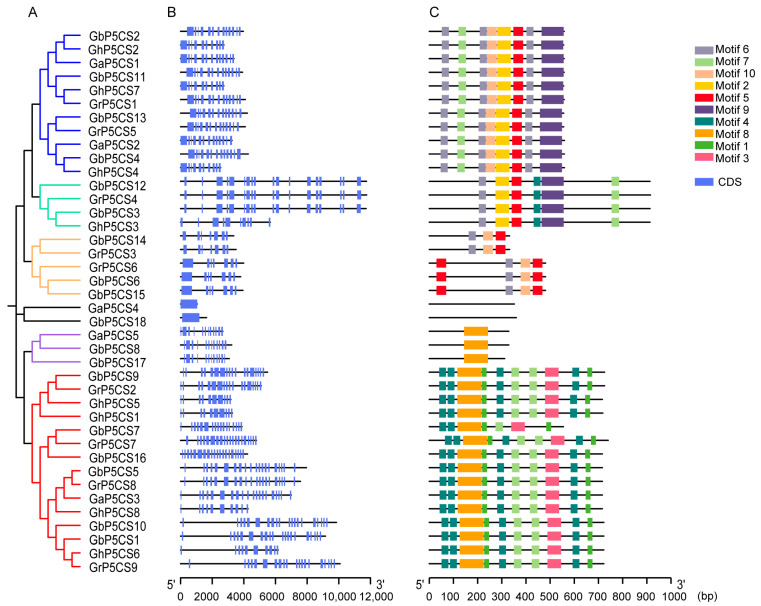
Gene structure and conserved motifs of *P5CS* genes across four cotton species. (**A**) Phylogenetic tree of the 40 *P5CS* genes identified within the four cotton species (**B**) Schematic representation of the exon–intron structure of *P5CS* genes. (**C**) Analysis of conserved motifs within P5CS proteins, with 10 motifs identified across the 40 P5CS proteins, each represented by a distinct color. The length of each motif is proportional to its frequency within the proteins.

To elucidate the structural compositions of *P5CS* genes in cotton, we compared the DNA and cDNA sequences of each gene to deduce their exon–intron structures (Figure 3B). The results revealed that a majority of *P5CS* genes possess complex structures, with the number of exons ranging from 7 to 20, except for two genes that contain only one intron. A significant proportion of *P5CS* genes (82.5%, 33/40) presented open reading frames with at least ten exons. Notably, all eight *GhP5CS* genes share the same structure, comprising ten exons, whereas the other three cotton species present varying exon numbers. Furthermore, our observations indicated that *P5CS* genes that are closely related in the evolutionary tree tend to have more similar exon and intron arrangements (Figure 3A,B). These findings suggest a strong correlation between exon–intron structure and phylogenetic relationships within this gene family.

To further delineate the characteristic regions of the P5CS proteins in cotton which are closely associated with their functions, we employed MEME v5.5.5 software to predict ten conserved motifs, designated as Motif 1 to Motif 10, among the P5CS proteins (Figure 3C). Two P5CS proteins, GbP5CS18 and GaP5CS4, were found to lack any motifs. The number of conserved motifs identified in the *P5CS* genes ranged from one to six. Interestingly, the distribution of these motifs was closely associated with the branches of the evolutionary tree. Motifs 2, 5, 6, 7, 9, and 10 were found in 11 *P5CS* genes within the blue clade; Motifs 2, 4, 5, 6, 7, and 9 were located in 4 *P5CS* genes within the light green clade; Motifs 1, 3, 4, 7, and 8 were detected in 15 *P5CS* genes within the red clade; and 5 *P5CS* genes in the orange clade contained Motifs 5, 6, and 10. In contrast, three *P5CS* genes in the purple clade possessed only Motif 8. These findings suggest that *P5CS* genes in different evolutionary clades exhibit distinct motif compositions and gene structures, indicating potential functional differentiation of *P5CS* genes in cotton. The variation in the distribution of conserved motifs may imply that these genes have evolved distinct functions within the cotton *P5CS* gene family.

### 2.4. Analysis of the Subcellular Localization of the P5CS Proteins in Cotton

Understanding the expression sites of proteins is crucial for exploring their functions and interaction patterns. Therefore, we predicted the expression locations of *P5CS* genes in cotton. The results revealed that almost all P5CS proteins across the four cotton species were expressed primarily in chloroplasts, mitochondria, and cytoplasm (Supplementary Figure S4), with no significant differences in expression sites observed. Specifically, 11 *P5CS* genes in *G. hirsutum*, *G. barbadense*, and *G. raimondii* were predicted to be located mainly in mitochondria. Nineteen genes were found to be predominantly localized to chloroplasts, with four, eight, four, and three genes in *G. hirsutum*, *G. barbadense*, *G. raimondii*, and *G. arboretum*, respectively. In addition, six *P5CS* genes were predicted to be expressed primarily in the cytoplasm. Furthermore, two genes in *G. barbadense* and one *P5CS* gene in *G. raimondii* were most likely expressed in the nucleus (Supplementary Figure S4A–D). These findings suggest that *P5CS* genes may play diverse roles at different cellular sites, primarily related to the metabolic pathways of proline in mitochondria and chloroplasts.

### 2.5. Cis-Acting Element Analysis of the P5CS Gene Promoters in Cotton

To further investigate the potential functions of the *P5CS* genes in the stress response, the 1.5 kb promoter sequence upstream of each *P5CS* gene in the four cotton species was isolated and analyzed to identify potential *cis*-acting regulatory elements (CAREs). A total of 18 CAREs within the promoter regions of *P5CS* genes were identified (Supplementary Figure S5). These elements contained five stress-responsive elements, including MBSs (drought-induced MYB binding site elements), LTRs (low-temperature response elements), CAAT-box and ABRE (salt and drought response elements), GT1 (salt response elements), and WUN-motif (wounding induction elements), as well as eight hormone-responsive elements, such as ABREs, AREs, CGTCA-motif, GARE-motif, MYB, TGACG-motif, P-box, and TCA-element. The MYB *cis*-acting elements, which are involved in controlling plant secondary metabolism, regulating cell morphogenesis, and responding to environmental factors, were found in most of the *P5CS* genes. Notably, the traditional promoter element, the CAAT-box, was present in all *P5CS* promoters. This element is involved in the regulation of gene expression in plants under different environmental stresses, such as drought, salinity, and cold, and is also involved in normal growth and development. Under stress conditions, the CAAT-box may interact with ABRE (related to ABA), CGTCA-motif (related to methyl jasmonate), and P-box (gibberellin) elements to regulate downstream gene expression, thereby adapting to various stresses. These findings strongly suggest that cotton *P5CS* genes may play a role in mediating responses to abiotic stresses.

### 2.6. Secondary Structure Prediction and Three-Dimensional Modeling of the GhP5CS Proteins

Since the close relationship between protein structure and function, we further predicted the secondary and tertiary structures of eight *GhP5CS* genes in *G. hirsutum* to increase our understanding of their roles. The secondary structure predominantly consists of α-helices and random coils, followed by extended strands and β-turns (Supplementary Table S4). Also, α-helices were the most abundant, constituting 39.57% to 50.70% of the secondary structure, followed by random coils (26.40% to 38.13%), extended strands (approximately 16%), and β-turns (approximately 6%).

The three-dimensional structures of the GhP5CS proteins were modeled via homology modeling (Supplementary Figure S6). These models facilitated clear visualization of the composition and positions of secondary structures within GhP5CS proteins. The secondary structure elements, α-helices, random coils, extended strands, and β-turns further fold into a compact globular spatial structure through interactions between side chain groups and the maintenance of various secondary bonds. Notably, two distinct types of three-dimensional models were identified, which exhibited significant structural differences. The three-dimensional conformations of GhP5CS1, GhP5CS5, GhP5CS6, and GhP5CS8 are similar, whereas the remaining four GhP5CS proteins exhibit another set of similar three-dimensional structures. These observations suggest potential functional differentiation among GhP5CS proteins and imply that they may participate in different biological processes.

### 2.7. Expression Patterns of GhP5CS Genes Under Salt Stress

**Figure 4:**
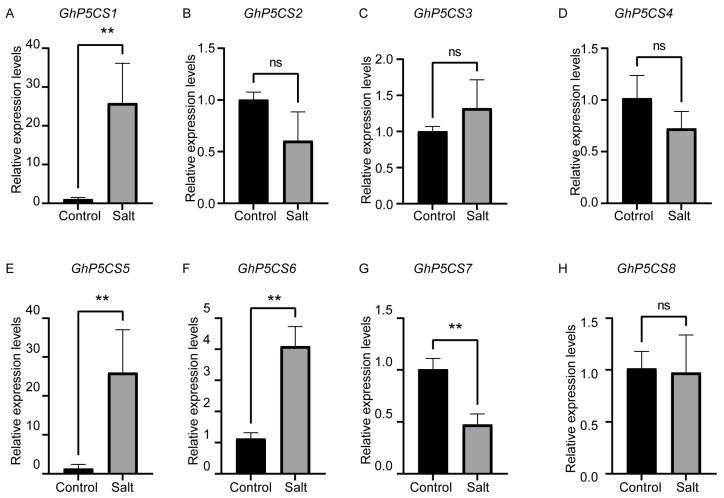
qRT-PCR analysis of the expression of eight *GhP5CS* genes, *GhP5CS1* to *GhP5CS8* (**A**–**H**), respectively, in cotton leaves under salt stress conditions. Data are represented as mean ± standard error (SE) from three biological replicates. The symbols ** indicate significant differences at *p* < 0.01, while ‘ns’ means not significant. Statistical analysis was performed using Student’s *t*-test to compare the relative expression of *GhP5CS* genes between salt-stressed and control groups.

**Figure 5:**
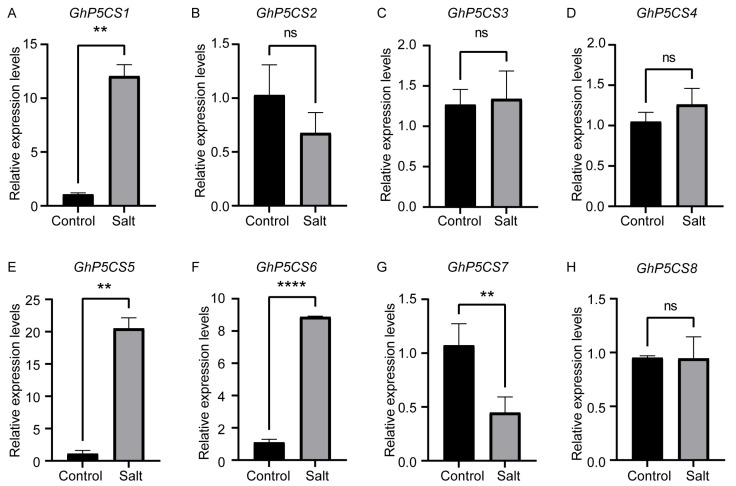
qRT-PCR analysis of the expression of eight *GhP5CS* genes, *GhP5CS1* to *GhP5CS8* (**A**–**H**), respectively, in cotton roots under salt stress conditions. Data are represented as mean ± standard error (SE) from three biological replicates. The symbols ** indicate significant differences at *p* < 0.01 and symbols **** indicate significant differences at *p* < 0.0001, respectively, while ‘ns’ means not significant. Statistical analysis was performed using Student’s *t*-test to compare the relative expression of *GhP5CS* genes between salt-stressed and control groups.

Previous studies have established that *P5CS* genes play pivotal roles in abiotic stress response in various plants, supported by the presence of the stress-responsive elements in their promoter regions. To explore the molecular function of the *GhP5CS* genes under salt stress, we assessed the expression levels of all eight *GhP5CS* genes in the leaves and roots of upland cotton TM-1 plants under control conditions and salt stress using qRT-PCR. Under salt stress, four out of the eight *GhP5CS* genes exhibited significant differences in expression in both leaves and roots, namely *GhP5CS1*, *GhP5CS5*, *GhP5CS6*, and *GhP5CS7*, suggesting their potential role in response to salt stress, while the other four genes did not (Figure 4 and Figure 5). Among the four genes whose expressions significantly differed, the expression of *GhP5CS7* was downregulated in both the leaves and roots, whereas that of the remaining genes was upregulated under salt stress. Furthermore, *GhP5CS1*, which presented the greatest increase in expression under salt stress (23.9-fold in leaves and 11.6-fold in roots), may be the key regulatory gene involved in the response to salt stress in cotton. Collectively, these results indicate that some *GhP5CS* genes are indeed involved in the salt stress response of cotton, but they may operate through different mechanisms due to their distinct expression patterns.

### 2.8. Virus-Induced Gene Silencing of GhP5CS1 Leads to Salt Sensitivity in Cotton

**Figure 6:**
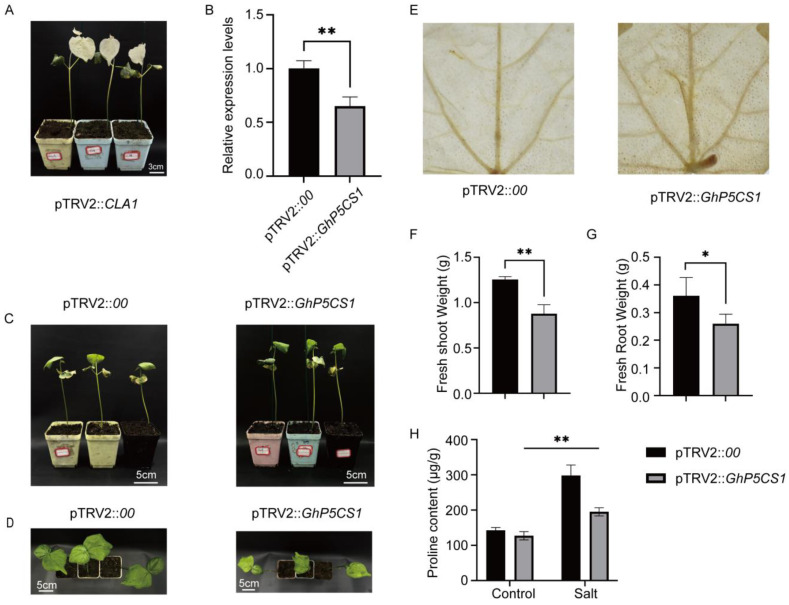
Functional validation of *GhP5CS1* under salt stress using VIGS. (**A**) The albino phenotype observed in newly emerged true leaves of the pTRV2::*CLA1* plants two weeks post-injection. (**B**) Relative expression levels of *GhP5CS1* in pTRV2::*00* and pTRV2::*GhP5CS1* plants, sampled before salt treatment. (**C**) Appearance of pTRV2::*00* and pTRV2::*GhP5CS1* plants before salt stress treatment. (**D**) Significant differences in leaf size between pTRV2::*00* and pTRV2::*GhP5CS1* plants after salt treatment for ten days. (**E**) DAB staining of leaves from pTRV2::*00* and pTRV2::*GhP5CS1* plants after one week of salt treatment. (**F**) The fresh weight of shoots from pTRV2::*00* and pTRV2::*GhP5CS1* plants after two weeks of salt treatment. (**G**) The fresh weight of roots from pTRV2::*00* and pTRV2::*GhP5CS1* plants after two weeks of salt treatment. (**H**) Proline content in leaves of pTRV2::*00* and pTRV2::*GhP5CS1* plants under control and salt conditions after three weeks of salt treatment. The symbols * and ** indicate significant differences at *p* < 0.05 and *p* < 0.01, respectively. Student’s *t*-test was used to analyze the differences in the relative expression of *GhP5CS1* gene between pTRV2::*00* and pTRV2::*GhP5CS1* plants under salt treatment and control conditions.

Recognizing the pronounced upregulation of *GhP5CS1* under salt stress, as confirmed by qRT-PCR, we used Virus-Induced Gene Silencing (VIGS) technology to downregulate its expression in upload cotton to definitively establish the role of *GhP5CS1* in response to salt stress in cotton. The pTRV2::*CLA1* plants, which served as a positive control, developed an albino phenotype in newly emerged true leaves, thereby validating the efficacy of our experimental system (Figure 6A).

Subsequently, we suppressed the expression of *GhP5CS1* using the same VIGS system by constructing the pTRV2::*GhP5CS1* fusion vector. Samples of young leaves from both pTRV2::*00* and VIGS plants were collected, and qRT-PCR analysis confirmed a significant decrease in the expression levels of VIGS plants, indicative of successful gene silencing (Figure 6B). No significant differences in growth were detected between the qTRV2::*00* and VIGS plants under normal conditions (Figure 6C). However, under salt stress, the growth of the *GhP5CS1*-silenced plants was markedly slower, and the size of their true leaves was considerably smaller than that of the control plants (Figure 6D). Additionally, compared with the control plants, the gene-silenced plants displayed more severe oxidative damage upon DAB staining (Figure 6E), suggesting increased susceptibility to salt stress-induced damage. The fresh weights of both the roots and shoots also noticeably differed between the gene-silenced and control plants, demonstrating that salt stress substantially hindered plant growth when the *GhG5PS1* gene was silenced (Figure 6F,G). Moreover, the proline content in the gene-silenced plants was significantly lower than that in the control plants, further indicating the involvement of *GhP5CS1* in proline biosynthesis, a key osmolyte that confers salt tolerance in cotton (Figure 6H). This reduction in proline accumulation in the silenced plants likely underpins their increased sensitivity to salt stress. Collectively, these findings confirm that *GhP5CS1* plays a pivotal role in the response of cotton to salt stress, potentially by modulating proline biosynthesis.

## 3. Discussion

Many plant species have undergone a genome-wide replication event, known as polyploidy [29], which resulted in extensive chromosome doubling and the retention of numerous repetitive chromosomal fragments. Specifically, *G. hirsutum* and *G. barbadense* are identified as allotetraploids, having arisen from the hybridization of two distinct cotton species, with *G. arboretum* and *G. raimondii* serving as the genomic contributors to *G. hirsutum* [30,31,32,33,34]. In our analysis, we identified eight *P5CS* gene members in *G. hirsutum*, eighteen in *G. barbadense*, five in *G. arboretum*, and nine in *G. raimondii* (Figure 1). Notably, the number of *P5CS* genes in *G. hirsutum* does not correspond to the cumulative total of its progenitors, indicating that gene loss occurs to varying extents during the process of polyploidization [34]. Furthermore, the *P5CS* gene count in *G. hirsutum* is lower than that in *G. barbadense*, suggesting that chromosome doubling and rapid genomic rearrangements contribute to gene loss [35].

A phylogenetic analysis of *P5CS* genes across the four cotton species revealed the presence of three distinct subgroups, with certain genes from *A. thaliana* and *T. cacao* exhibiting close phylogenetic relationships with the dicotyledonous cotton plants (Figure 1). These observations imply that *A. thaliana* and *T. cacao* may serve as valuable reference points for *P5CS* gene research in cotton, reinforcing the hypothesis of a shared common ancestor between *T. cacao* and cotton [30,36]. Gene localization studies indicated similar distribution patterns of *P5CS* genes in *G. hirsutum* and *G. barbadense* (Supplementary Figure S1), further supporting the concept of a common ancestry.

The presence of duplicate genes on the same chromosome is likely attributable to tandem repeats, while those exhibiting high sequence similarity across different chromosomes are primarily the result of segmental duplications [37]. Tandem duplications, which predominantly occur in regions of chromosomal recombination, give rise to gene clusters with analogous sequences and functions [38]. Fragment replication, which often leads to dispersed duplicated genes, plays a significant role in the evolution of organisms, with many plants displaying multiple duplicated chromosomal blocks [39]. In our investigation, tandem repeats were exclusively observed on chromosomes A11 and D11 of *G. barbadense*, as well as on chromosome 7 of *G. raimondii*, each containing two genes (Supplementary Figure S1). These duplications belong to the same subfamily within the evolutionary tree, suggesting closer evolutionary relationships and functional similarities. However, the limited occurrence of tandem repeats indicates a relatively minor contribution to the expansion of *P5CS* gene family in cotton.

Additionally, analyses of collinearity and selection pressure analysis revealed multiple pairs of duplicate genes among different cotton species, particularly between the tetraploid *G. hirsutum* and *G. barbadense* (Figure 2A), likely as a consequence of their shared allopolyploidy events. During the process of polyploidization, tetraploid genomes expand gene families derived from diploid genomes (A and D) [31]. Similar amplifications have been documented in the *Erf* [40] and *NFYA* [41] gene families within cotton. We propose that extensive fragmental duplications and genomic polyploidy represent the primary mechanisms for the formation of *P5CS* genes in cotton. Collinearity analysis further indicated minimal relationships among *G. hirsutum*, *G. arboretum*, and *G. raimondii* (Figure 2B,C and Supplementary Figure S3), suggesting that most *P5CS* genes have been involved independently. The KA/KS analysis demonstrated that all the gene pairs exhibited KA/KS values significantly less than 1 (Supplementary Table S4), indicating rapid evolution following duplication [42]. This genetic diversity enhances cotton’s ability to regulate complex traits, thereby augmenting its evolutionary and adaptive potential. These findings are pivotal for advancing our understanding of the genetics, evolution, and breeding of cotton.

In *A. thaliana*, two *P5CS* genes exhibit distinct functional roles, with *P5CS1* being critical for proline accumulation in response to osmotic stress, while *P5CS2* is implicated in seed development, indicating a divergence in function. In *G. hirsutum*, evidence suggests a similar divergency among the *P5CS* genes, as indicated by their differential expression patterns under salt stress (Figure 4 and Figure 5). Specifically, qRT-PCR analysis revealed that four out of the eight *GhP5CS* genes were significantly upregulated in response to salt stress, whereas the remaining genes exhibited no change in expression. Moreover, the four upregulated *GhP5CS* genes appeared to have more *cis*-acting elements related to stress response, such as ABRE and GT-1 (Supplementary Figure S5), which might be one of the reasons for their differences in salt stress response. These observations imply a functional diversity among *P5CS* genes and highlight their critical role in plant stress responses. Additionally, the regulatory functions of *P5CS* genes in various crops have been documented in the literature. Consequently, based on the qRT-PCR findings, we employed the VIGS technology to silence the *GhP5CS1* gene, which demonstrated the most significant upregulation under salt stress. This intervention resulted in a marked reduction in *GhP5CS1* expression, delayed growth, and increased oxidative damage in the gene-silenced plants compared to the control plants (Figure 6A–G). Furthermore, the proline content in the gene-silenced plants decreased under salt stress, indicating a compromised ability to accumulate proline when *GhP5CS1* expression was inhibited (Figure 6H). These results not only enhance our understanding of the functional diversity of the *P5CS* genes in cotton but also emphasize the potential significance of *GhP5CS1* in the plant’s response to salt stress. Moreover, these findings provide a robust foundation for the molecular breeding of the *GhP5CS1* gene aimed at developing salt-tolerant cotton germplasm.

In the evolution of many plants, functional redundancy is prevalent among many paralogous genes, potentially arising from gene replication [43]. It boosts adaptability and robustness by compensating for gene mutations or environmental changes, and it also facilitates evolutionary innovation through the potential for new functions and specialization [44]. In *A. thaliana*, *ABCB6* and *ABCB20* showed functional redundancy in auxin transport, and their combined loss of function led to significant developmental defects [45]. Additionally, several members of the *MIR172* gene family share overlapping functions in regulating specific aspects of plant development [46]. In this study, *GhP5CS5* and *GhP5CS6* shared the same salt-induced expression pattern as *GhP5CS1*. Downregulating *GhP5CS1* expression resulted in salt sensitivity in cotton, indicating that *GhP5CS1* had an independent response to salt stress. However, *GhP5CS5* and *GhP5CS6* may exhibit functional redundancy, which requires further genetic transformation evidence for confirmation. Conversely, *GhP5S7* has an opposite salt-induced expression pattern compared to the three *GhP5CS* genes, suggesting potential functional differentiation, which also needs further verification.

## 4. Materials and Methods

### 4.1. Identification of P5CS Gene Members in Different Species

The genomic data for five species, including the tetraploid *Gossypium hirsutum* and *Gossypium barbadense* and the diploid *Gossypium raimondii*, as well as *T. cacao* and *Z. mays*, were retrieved from the Phytozome database (https://phytozome-next.jgi.doe.gov/ accessed on (8 September 2023)). For the diploid *G. arboretum*, the CRI genome database was obtained via the CottonFGD (https://cottonfgd.net/ accessed on (8 September 2023)). The genomic data for *A. thaliana*, *O. sativa*, and *S. bicolor* were sourced from the Ensembl Plants database (http://plants.ensembl.org/index.html/ accessed on (8 September 2023)). These databases include coding sequences (CDSs), gene annotation files, and protein sequences.

We employed a hidden Markov model (HMM) to query the P5CS protein sequences harboring the PPbinding domain (PF00696) in Pfam (http://pfam.xfam.org/ accessed on (10 September 2023)) across the various genomes via default parameters in HMMER v3.4 software [47,48,49]. The Hummsearch program running on a Linux system was used to identify proteins with the conserved domain, setting an E-value threshold at 1 × 10^−20^. The candidate protein sequences were then submitted to SMART (http://smart.embl.de/ accessed on (20 September 2023)), Pfam (http://pfam-legacy.xfam.org/ accessed on (20 September 2023)), and NCBI CDD (https://www.ncbi.nlm.nih.gov/cdd/ accessed on (20 September 2023)) for validation [50,51]. The amino acid length and isoelectric point (PI) of the identified proteins were calculated via the ExPASy proteomics server (http://www.expasy.org/ accessed on (22 September 2023)) [52].

### 4.2. Chromosome Localization Analysis of P5CS Genes in Cotton Species

Genomic annotation data were utilized to ascertain the chromosome length and positional information of all *P5CS* genes. MapChart v2.32 software [53] was then employed to create a chromosomal localization map for the *P5CS* genes across the four cotton species, which were subsequently refined and archived via Adobe Illustrator CS6 (Adobe Inc., San Jose, CA, USA).

### 4.3. Phylogenetic Analysis, Collinearity, and KA/KS Ratios of P5CS Genes

A phylogenetic tree of P5CS proteins from nine species was constructed using the neighbor-joining method, following multiple sequence alignment with MEGA 7.0 software [54]. Branch support was assessed via a bootstrap test with 1000 iterations, and the evolutionary tree was further edited via the Evolview online platform [55]. The collinear gene pairs were identified through BLAST comparisons of P5CS protein sequences with the Multiple Collinearity Scan Toolkit (MCScanX) [56].

KA, KS, and their ratios were determined to evaluate evolutionary selection pressures on the genes. The Calculator tools were employed to determine the nonsynonymous (KA) and synonymous (KS) substitution rates in *P5CS* genes across four cotton species.

### 4.4. Gene Structure and Protein Conserved Motif Analysis of P5CS Genes in Four Cotton Species

We employed the Gene Structure Display Server (GSDS) (http://gsds.cbi.pku.edu.cn/ accessed on (10 October 2023)) to analyze the gene structure of *P5CS* genes on the basis of their CDS, 3′UTR, and 5′UTR sequences [57]. The MEME online platform (https://meme-suite.org/meme/ accessed on (12 October 2023)) was employed to identify conserved motifs within *P5CS* genes, with parameters set to a maximum of 10 motifs and an optimal motif width ranging from 6 to 50 residues [58]. TBtools v2.056 software was used to integrate and visualize the phylogenetic tree, gene structure, and conserved motif images of the *P5CS* genes.

### 4.5. Subcellular Localization Prediction of P5CS Proteins

The CELLO website (http://cello.life.nctu.edu.tw/ accessed on (21 October 2023)) was used to predict the subcellular localization of P5CS proteins by submitting their sequences. The results were then visualized via TBtools [59].

### 4.6. Cis-Acting Element Analysis of P5CS Gene Promoter Regions

Promoter sequences of 1.5 kb upstream of the transcription start site for each *P5CS* gene were extracted from four cotton species. The PlantCARE (*Cis*-acting Regulatory Elements, http://bioinformatics.psb.ugent.be/webtools/plantcare/html/ accessed on (29 October 2023)) online platform was used to identify the CAREs within these promoter regions [60]. Visual representations of these CAREs were generated via the GSDS online platform.

### 4.7. Secondary Structure Prediction and Three-Dimensional Model Construction of GhP5CS Proteins

The Prabi online platform (https://npsa-prabi.ibcp.fr/ accessed on (6 November 2023)) was used to predict the secondary structure of the GhP5CS proteins. For detailed structural analysis, three-dimensional models of GhP5CS proteins were constructed via a homology-based protein modeling approach by submitting their amino acid sequences to the SWISS-MODEL website (https://swissmodel.expasy.org/ accessed on (7 November 2023)) [61].

### 4.8. Plant Materials, Salt Treatment, RNA Extraction, and qRT-PCR Analysis

Seeds of the upland cotton line TM-1 were sterilized with 75% ethanol for 1 min and then soaked in sterile water for 6 h. The seeds were subsequently sown in pots with a mixture of nutrient soil and vermiculite (at a ratio of 3:1) and grown in a greenhouse. The temperature and light cycle were set at 26 °C, with a 14 h/10 h (light and dark) photoperiod. Once the second true leaf fully expanded, the seedlings were transferred to Hoagland nutrient solution and acclimated for three days. The samples were then divided into control and experimental groups, with the latter receiving a 200 mmol/L NaCl solution. After two days of treatment, the fresh leaves and roots of both groups were collected, with three biological replicates per sample, each consisting of three cotton seedlings mixed together. The samples were ground in liquid nitrogen immediately after sampling, and the remaining samples were stored in a −80 °C freezer. Total RNA was then extracted from the leaves and roots using a RNAprep pure plant plus kit for polysaccharide and polyphenols (Tiangen, Beijing, China), with each sample weighing about 100 mg, and the total RNA’s integrity was assessed via 1.2% agarose gel electrophoresis. RNA concentrations were measured using a Nanodrop2000 microspectrophotometer (Thermo Fisher Scientific, Waltham, MA, USA). One milligram of total RNA for each sample was immediately used to synthesize the first-strand cDNA using the HiScript III RT SuperMix for qPCR kit (+gDNA wiper) (Vazyme, Nanjing, China). The remaining RNA was stored in a medical refrigerator at −20 °C. The gene-specific primers for RT-qPCR analysis were designed using Primer 5.0 software, and the primer sequences are listed in Supplementary Table S5. The forward and reverse primers of each *GhP5CS* gene, ranging from 20 to 22 bp in length, were designed to target different exons to exclude genomic DNA amplification, and their amplified products ranged from 100 to 250 bp. The cDNA was diluted ten-fold and used as a template for the RT-qPCR reaction, with 1 μL used per reaction. RT-qPCR was performed in triplicate for each biological replicate to ensure accuracy, using ChamQ SYBR qPCR Master Mix (Low ROX Premixed) (Vazyme, Nanjing, China) with a SLAN-96S PCR system (Hongshi medical, Shanghai, China) to assess the expression levels of eight *GhP5CS* genes under the control and salt treatments. The parameters were set as follows: Stage 1, 95 °C for 30 s; Stage 2, 40 cycles of 95 °C for 10 s and 60 °C for 30 s; and Stage 3, melting curve. The cotton *Ubiquitin* gene served as the internal reference gene, and relative expression levels were calculated via the comparative threshold cycle method [62]. Data were presented as mean ± standard error (SE) and Student’s *t*-test was used for significance analysis, with *p* < 0:05 considered significant.

### 4.9. Virus-Induced Gene Silencing (VIGS)

The specific primers for the *GhP5CS1* gene were designed to amplify a 313 bp fragment for VIGS. The empty plasmid pTRV2::*00* was linearized using Sacl and BamHI restriction enzymes (NEB, Beverly, MA, USA). The pTRV2::*GhP5CS1* fusion vector was recombined via the ClonExpress^®^ II One Step Cloning Kit (Vazyme, Nanjing, China). The recombinant plasmid, along with pTRV2::*00* (negative control) and pTRV2::*CLA1* (positive control) was transformed into *Agrobacterium tumefaciens* (GV3101). After screening the positive clones, the bacterial solution was injected into the cotyledons of cotton seedlings using sterile syringes when they had fully expanded after about 10 days of cultivation. After a 48 h dark treatment, the seedlings were moved to an artificial climate chamber. The positive control exhibited an albino phenotype approximately two weeks later. Once the albino phenotype of positive control appeared, the negative control and silenced plants were treated with 400 mmol/L NaCl solution. Each pot of seedlings was watered every two days, with 50 mL per pot. After 10 days of treatment, the root and shoot fresh weights were measured for each plant.

### 4.10. DAB Staining and Proline Content Determination

DAB (3,3′-diaminobenzidine tetrachloride) staining was performed to locate hydrogen peroxide in the tissues via a DAB staining kit (Leagene, Beijing, China). The fully expanded true leaves of the seedlings (pTRV2::*00* and pTRV2::*GhP5CS1*) after one week of salt treatment were collected and stained with 1 mg/mL DAB for 12 h. After 21 days of salt treatment, true leaves were collected, and the proline content was determined via visible spectrophotometry methods using a proline (Pro) content assay kit (Solarbio, Beijing, China).

## 5. Conclusions

The *P5CS* genes in cotton exhibit a complex pattern of genomic organization, evolutionary relationships, and functional diversity. Evolutionary and structural analyses revealed potential divergence and differentiation among these genes. VIGS-mediated downregulation of *GhP5CS1* led to increased salt sensitivity and reduced proline content, confirming its key role in salt stress tolerance. These insights highlight the significance of *P5CS* genes in the adaptation of cotton to abiotic stress and lay the foundation for further functional characterization and application in enhancing stress tolerance in cotton.

## Data Availability

The datasets supporting the results of this article, mainly including the genomic sequences, are available from CottonFGD (https://cottonfgd.net/ accessed on (8 September 2023)), Phytozome website (https://phytozome-next.jgi.doe.gov/ accessed on (8 September 2023)), and Ensembl Plants database (http://plants.ensembl.org/index.html/ accessed on (8 September 2023)).

## Supplementary Materials

The following supporting information can be downloaded at https://www.mdpi.com/article/10.3390/plants14020231/s1, Figure S1: Chromosome localization of *P5CS* genes in four cotton species; Figure S2: Interchromosomal relationships of *P5CS* genes among four cotton species; Figure S3: Collinearity analysis of *P5CS* genes between *G. raimondii* and *G. arboretum*; Figure S4: Prediction of subcellular localization for P5CS proteins; Figure S5: Characterization of *cis*-acting elements in the promoter regions of *P5CS* genes across four cotton species; Figure S6: Three-dimensional structural modeling of eight GhP5CS proteins; Table S1: Physical, chemical properties, and subcellular location of P5CS genes; Table S2: Gene pairs in collinearity among four cotton species; Table S3: KA, KS, and their ratios of pairwise genes across four cotton species; Table S4: The secondary structure statistics of the GhP5CS genes; Table S5: Primer sequences used in this study.
