# Supplementary material for: Unveiling the Role of GhP5CS1 in Cotton Salt Stress Tolerance: A Comprehensive Genomic and Functional Analysis of P5CS Genes
Source: Plants (Basel). 2025 Jan 15;14(2):231. doi: 10.3390/plants14020231 (PMC11768395; doi:10.3390/plants14020231)
Supplement: Supplementary file 1 [file plants-14-00231-s001.zip › plants-3410963-supplementary/Supplementary figures_250115.pdf]

The Supporting Information: Figs. S1-S6 and Tables S1-S5

Article title: Unveiling the role of *GhP5CS1* in cotton salt stress tolerance: a comprehensive genomic and functional analysis of P5CS genes

Hui Fang, Xin Gao, Yunhao Wu, Ke Zhang, Ying Wu, Junyi Li, Dongmei Qian, Ruochen Li, Haijing Gu, Teame Gereziher Mehari, Xinlian Shen, Baohua Wang

The following Supporting Information is available for this article:

Figure S1. Chromosome localization of *P5CS* genes in four cotton species. Chromosomes are represented by vertical lines, numbered at the top. Cotton species are distinguished by color: green for *G. hirsutum*, orange for *G. barbadense*, pink for *G. arboreum*, and light blue for *G. raimondii*.

Figure S2. Interchromosomal relationships of *P5CS* genes among four cotton species. Grey lines represent all syntenic blocks across the genomes, while red lines highlight collinear blocks of P5CS genes.

Figure S3. Collinearity analysis of *P5CS* genes between *G. raimondii* and *G. arboretum*.

Figure S4. Prediction of subcellular localization for P5CS proteins. Circles vary in color and size to reflect the confidence levels of the prediction outcomes. P5CS protein identifiers are listed on the left, with their predicted subcellular localizations indicated at the bottom.

Figure S5. Characterization of *cis*-acting elements in the promoter regions of *P5CS* genes across four cotton species. Different *cis*-acting elements are color-coded for distinction. Gene names are shown on the left, with promoter sequence lengths depicted to scale for accurate comparison.

Figure S6. Three-dimensional structural modeling of eight GhP5CS proteins. Homology modeling techniques were employed to analyze and model the GhP5CS proteins using the SWISS-MODEL platform, providing insights into their structural properties.

Table S1. Physical, chemical properties, and subcellular location of *P5CS* genes.

Table S2. Gene pairs in collinearity among four cotton species.

Table S3. KA, KS, and their ratios of pairwise genes across four cotton species.

Table S4. The secondary structure statistics of the *GhP5CS* genes.

Table S5. Primer sequences used in this study.

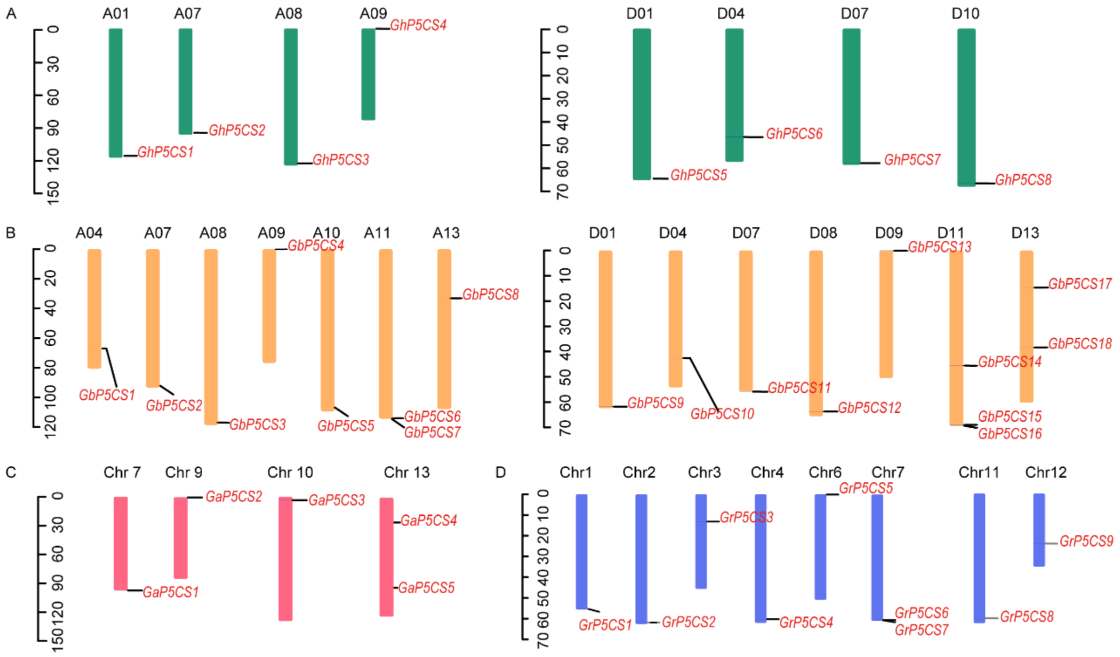

Figure S1. Chromosome localization of *P5CS* genes in four cotton species. Chromosomes are represented by vertical lines, numbered at the top. Cotton species are distinguished by color: green for *G. hirsutum*, orange for *G. barbadense*, pink for *G. arboreum*, and light blue for *G. raimondii*.

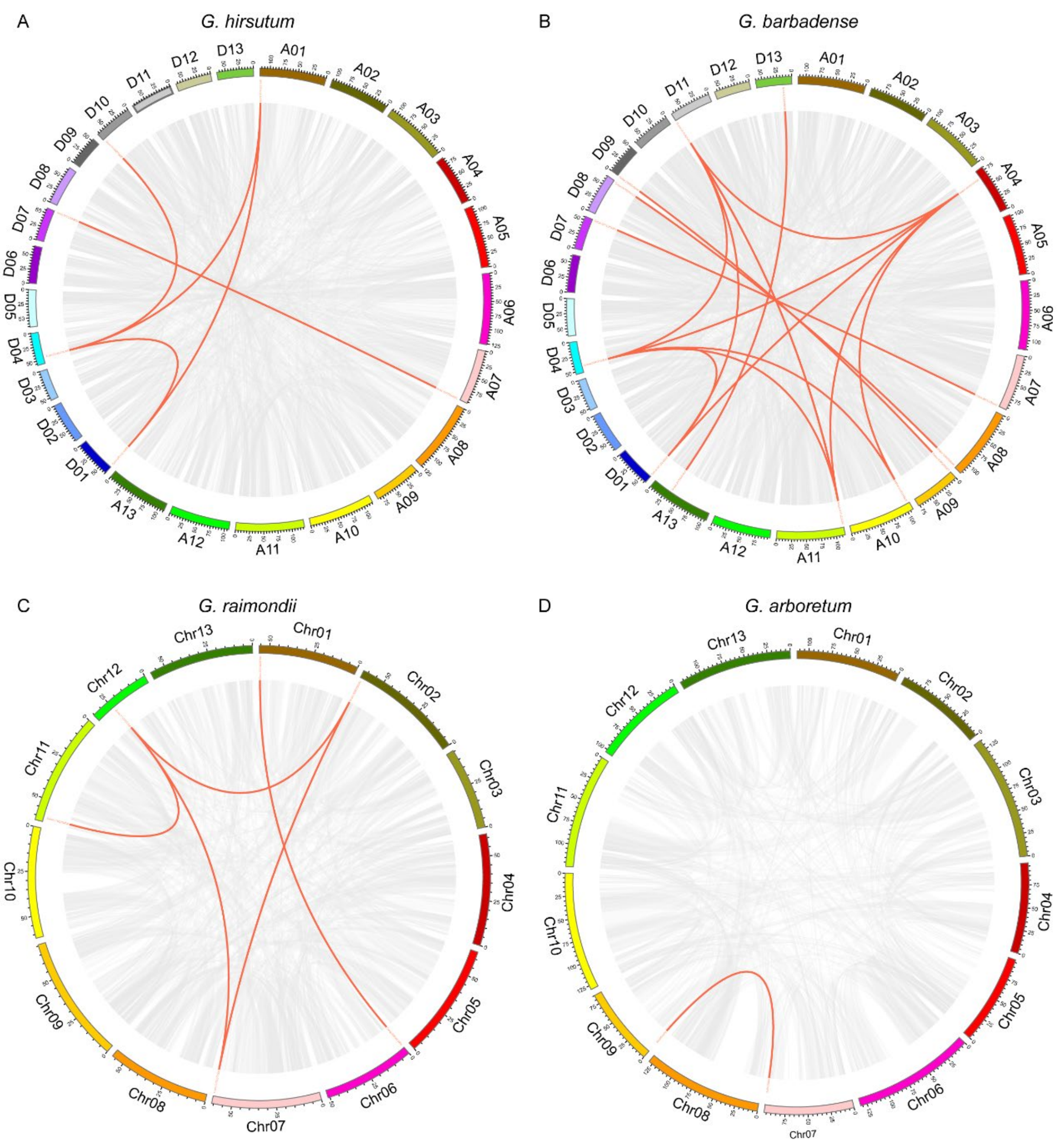

Figure S2. Interchromosomal relationships of *P5CS* genes among four cotton species. Grey lines represent all syntenic blocks across the genomes, while red lines highlight collinear blocks of *P5CS* genes.

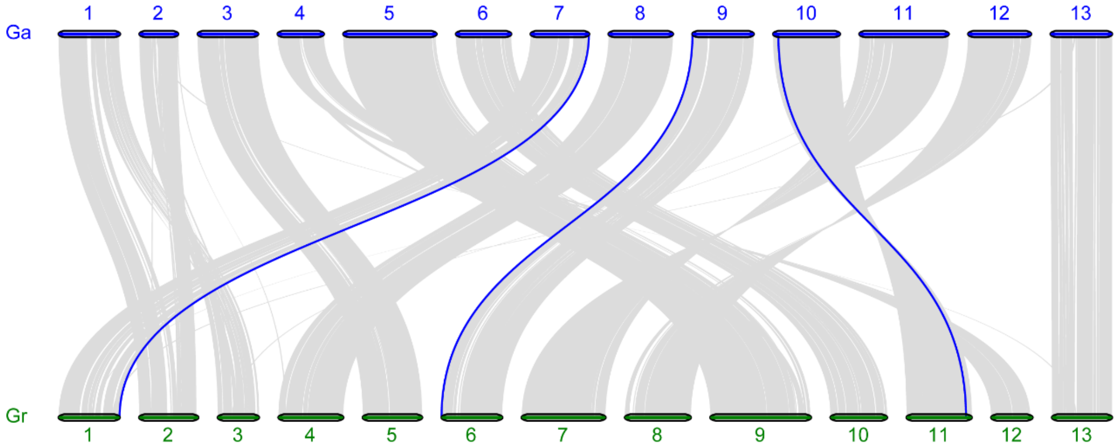

Figure S3. Collinearity analysis of *P5CS* genes between *G. raimondii* and *G. arboretum*.

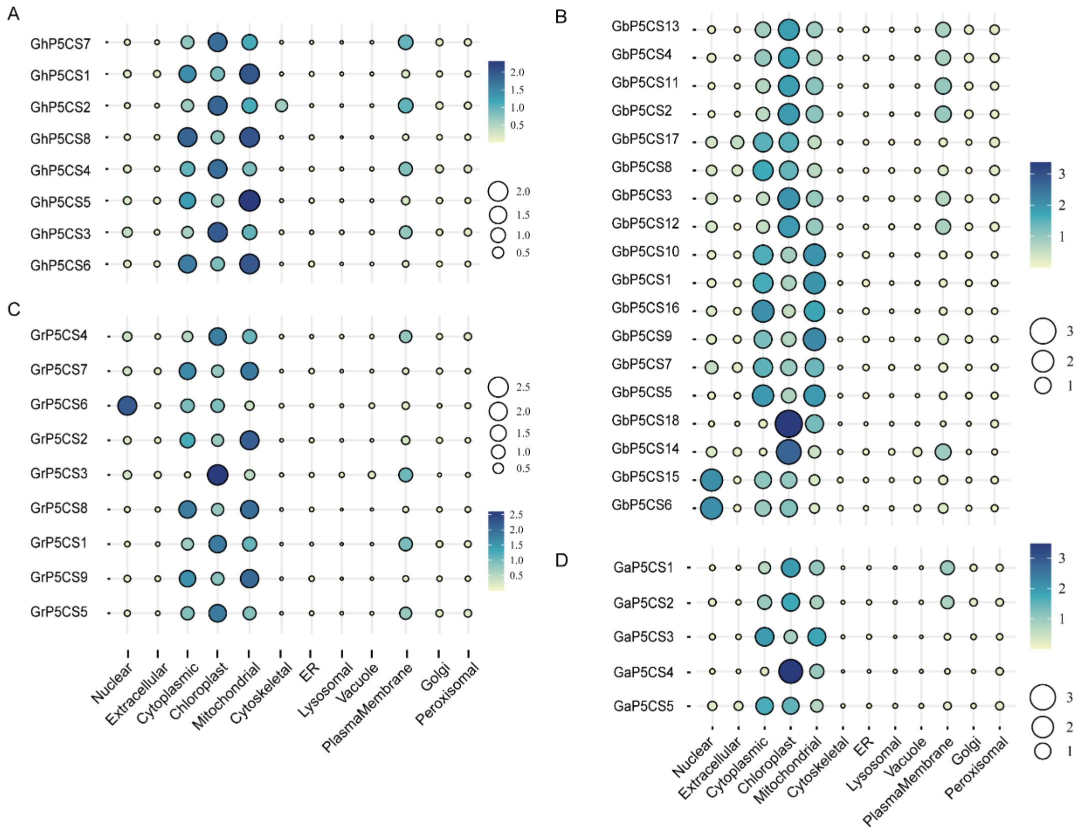

Figure S4. Prediction of subcellular localization for P5CS proteins. Circles vary in color and size to reflect the confidence levels of the prediction outcomes. P5CS protein identifiers are listed on the left, with their predicted subcellular localizations indicated at the bottom.

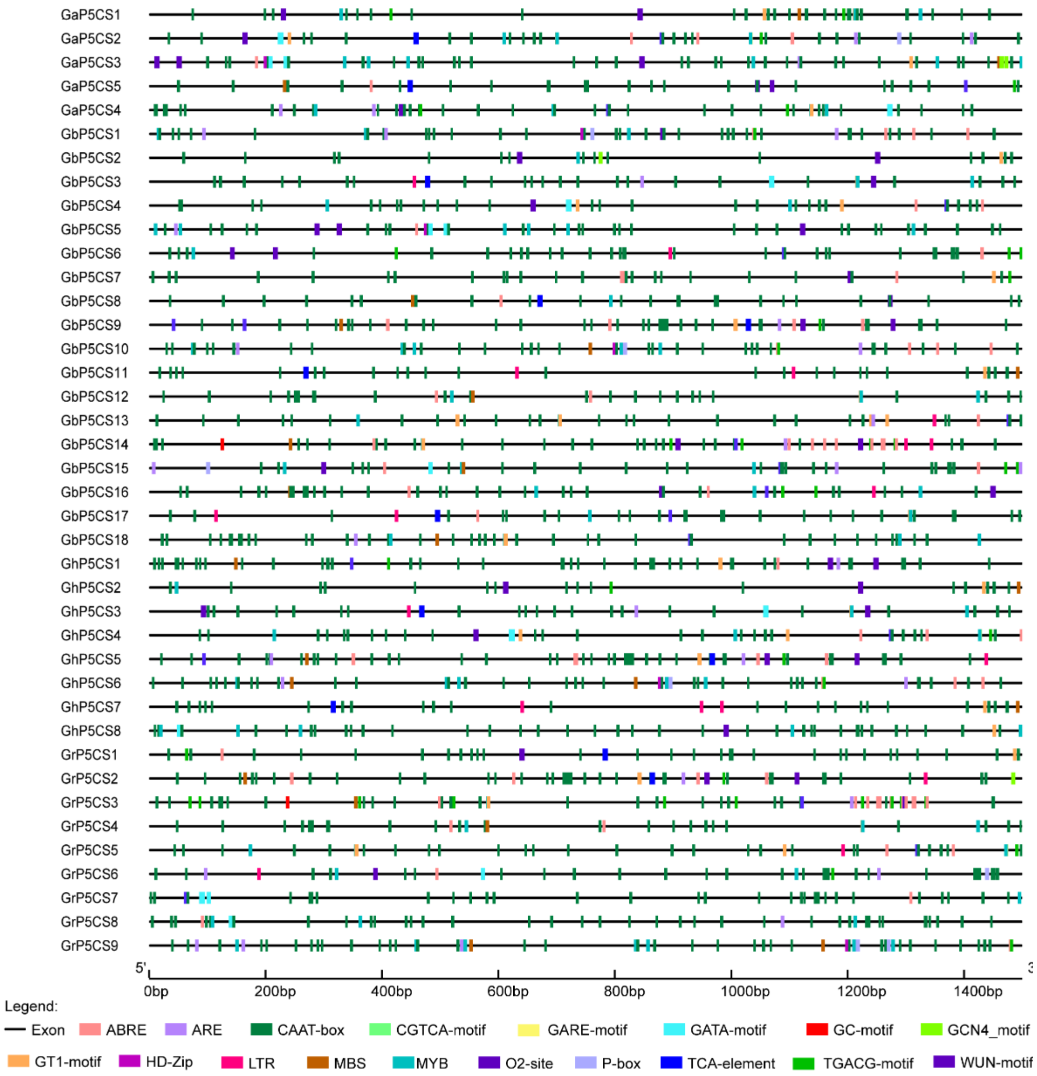

Figure S5. Characterization of *cis*-acting elements in the promoter regions of *P5CS* genes across four cotton species. Different *cis*-acting elements are color-coded for distinction. Gene names are shown on the left, with promoter sequence lengths depicted to scale for accurate comparison.

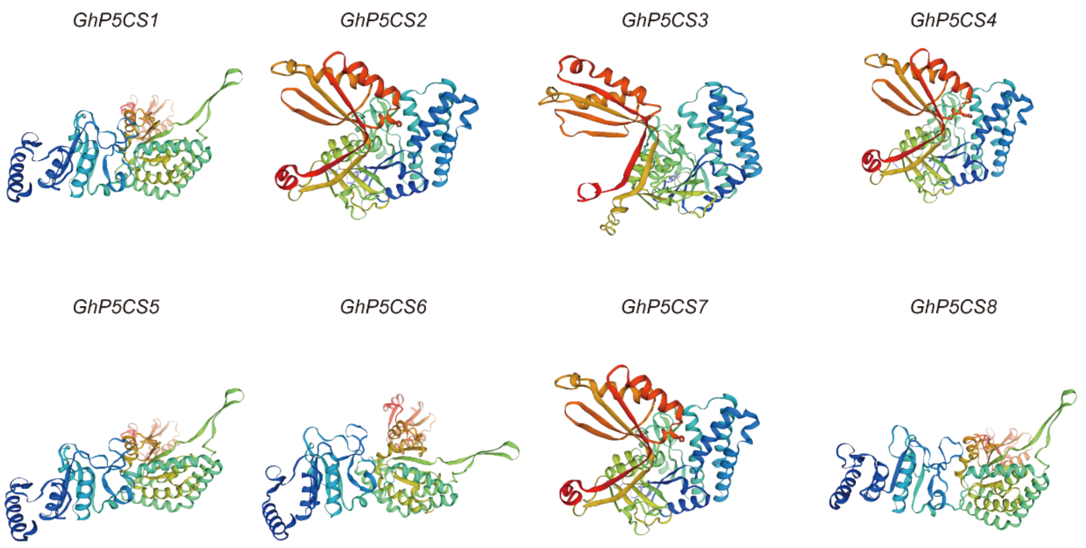

Figure S6. Three-dimensional structural modeling of eight GhP5CS proteins. Homology modeling techniques were employed to analyze and model the GhP5CS proteins using the SWISS-MODEL platform, providing insights into their structural properties.
